# Development of chiral metal amides as highly reactive catalysts for asymmetric [3 + 2] cycloadditions

**DOI:** 10.3762/bjoc.12.140

**Published:** 2016-07-13

**Authors:** Yasuhiro Yamashita, Susumu Yoshimoto, Mark J Dutton, Shū Kobayashi

**Affiliations:** 1 Department of Chemistry, School of Science, The University of Tokyo, Hongo, Bunkyo-ku, Tokyo, Japan

**Keywords:** [3 + 2] cycloaddition, asymmetric reaction, catalytic reaction, low catalyst loading, metal amide

## Abstract

Highly efficient catalytic asymmetric [3 + 2] cycloadditions using a chiral copper amide are reported. Compared with the chiral CuOTf/Et_3_N system, the CuHMDS system showed higher reactivity, and the desired reactions proceeded in high yields and high selectivities with catalyst loadings as low as 0.01 mol %.

## Findings

Catalytic asymmetric synthesis is an ideal method to prepare optically active compounds [1]. In this context, catalytic asymmetric carbon–carbon bond-forming reactions that can be used for the efficient construction of fundamental frameworks of complex chiral molecules such as biologically active compounds are particularly important. Chiral Lewis acid/Brønsted base-catalyzed carbon–carbon bond-forming reactions are one of the most efficient methods from the viewpoint of atom economy because only proton transfer occurs between starting materials and target products [2]. Several kinds of chiral Lewis acid/Brønsted base-catalyst systems have been developed; however, decreasing the catalyst loading is sometimes problematic either because of the low reactivity of catalysts or because the catalyst activity can be reduced through Lewis acid–Lewis base interaction between catalysts and the formed products (product inhibition). To overcome such issues, the design and development of more reactive catalysts is required.

Our group has focused on the development of metal amides as highly reactive Lewis acid/base catalysts in carbon–carbon bond-forming reactions [3]. Recently, we have developed asymmetric [3 + 2] cycloadditions [4–8] and asymmetric Mannich-type reactions [9] by using chiral silver or copper amides as catalysts. In these reactions, it has been revealed that the metal amides have higher activity than typical silver or copper acid/base catalysts, and that less reactive substrates react smoothly to afford the desired products in high yields with high stereoselectivities. Based on these results, it was considered that metal amide catalysts might also achieve high catalyst turnover. Here, we report chiral copper amide-catalyzed asymmetric [3 + 2] cycloadditions of Schiff bases of glycine ester that proceed with low catalyst loadings (ca. 0.01 mol %).

Catalytic asymmetric [3 + 2] cycloadditions of Schiff bases of α-amino esters to olefins are useful for synthesizing optically active pyrrolidine derivatives [10–12], and many highly stereoselective reactions have been reported; for example, Co [13], Cu [14–23], Ag [24–32], Zn [33–34], Ni [35–36], and Ca [37–39] catalyst systems, and organocatalysts [40–45] have been successfully employed. In most cases, however, relatively high catalyst loadings (0.5–25 mol %) are required to achieve high yield and selectivities [15,45]. First, we investigated the catalytic asymmetric [3 + 2] cycloaddition of Schiff base **1a**, prepared from glycine methyl ester and benzaldehyde, with *N*-phenylmaleimide (**2a**) in the presence of CuN(SiMe_3_)_2_ (CuHMDS) and the FeSulphos ligand, with the latter being related to the system reported by Carretero et al. [15]. The reaction produced **3aa** smoothly with 3 mol % catalyst loading at −40 °C, and high *endo* selectivity and high enantioselectivity were obtained (Table 1, entry 1). On the other hand, application of CuOTf, FeSulphos, and Et_3_N gave only 47% yield of the product (Table 1, entry 2). This result indicated that the CuHMDS catalyst had higher catalyst activity than CuOTf with the additional amine base. The copper amide catalyst also showed high reactivity and selectivity with 1 mol % catalyst loading (Table 1, entry 3), and similar results were obtained in other solvents such as Et_2_O and toluene, although the reactivity and enantioselectivity both decreased slightly in dichloromethane (DCM, Table 1, entries 4–6). It was found that the use of the chiral CuHMDS catalyst also afforded the product with high enantioselectivity at lower catalyst loadings of 0.1 mol % (Table 1, entry 7). The effect of the amide part of the structure was then examined. Copper dialkylamides were not as reactive as CuHMDS, and lower yields were obtained (Table 1, entries 8 and 9). Interestingly, mesitylcopper also worked in a similar fashion, and good yields and high selectivities were obtained (Table 1, entry 10). This result indicated that the reaction proceeded through a product base mechanism [46–48]; however, the reactivity was lower than that of the CuHMDS system. Decreasing the catalyst loading further revealed that the reaction proceeded with 0.01 mol % loading of chiral CuHMDS catalyst without significant loss of selectivity (Table 1, entry 11).

**Table 1:** Chiral copper amide-catalyzed asymmetric [3 + 2] cycloadditions^a^.

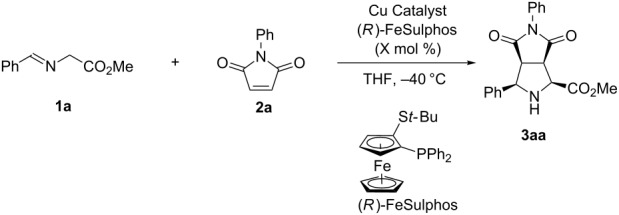							

Entry	Cu catalyst	Solvent	X	Time (h)	Yield (%)	*endo*/*exo*	ee (%, *endo*)

1	CuHMDS	THF	3	6	99	>99:1	99
2^b^	CuOTf + Et_3_N	THF	3	6	47	98:2	99
3^c^	CuHMDS	THF	1	6	98	>99:1	>99
4^c^	CuHMDS	Et_2_O	1	6	91	99:1	98
5^c^	CuHMDS	toluene	1	6	95	99:1	99
6^c^	CuHMDS	DCM	1	6	67	99:1	93
7^d^	CuHMDS	THF	0.1	18	94	>99:1	96
8^d^	CuNMe_2_^e^	THF	0.1	18	53	>99:1	94
9^d^	CuTMP^e^	THF	0.1	18	46	>99:1	98
10^d^	Cu(mesityl)	THF	0.1	18	86	>99:1	97
11^f^	CuHMDS	THF	0.01	48	94	>99:1	95

^a^The [3 + 2] cycloaddition reaction of 0.5 M **1a** (0.30 mmol) with **2a** (1.1 equivalents, 0.33 mmol) were conducted at −40 °C in the presence of the copper amide prepared from CuOTf·0.5toluene complex/KHMDS/FeSulphos (1.1:1.0:1.1) in situ unless otherwise noted. ^b^CuOTf·0.5toluene complex (0.0090 mmol) and Et_3_N (0.0090 mmol) were used. ^c^The reaction was conducted with **1a** (1.0 mmol). ^d^The reaction was conducted with **1a** (10 mmol). ^e^The copper amides were prepared in situ by mixing CuOTf·0.5toluene complex, FeSulphos and LiNMe_2_ or lithium 2,2,6,6,-tetramethylpiperidide (LiTMP). ^f^The reaction was conducted with 1.25 M **1a** (50 mmol).

We then examined the substrate scope of the [3 + 2] cycloaddition with respect to the Schiff base (Table 2). The Schiff bases prepared from tolualdehydes were successfully employed in the reaction with **2a**, and high reactivities and enantioselectivities were observed by using 0.1 mol % catalyst loading (Table 2, entries 1–4). The Schiff base from *p*-methoxybenzaldehyde was a good substrate (Table 2, entry 5) and reacted even in the presence of 0.01 mol % catalyst loading, albeit with a slight decrease in the enantioselectivity (Table 2, entry 6). The use of Schiff bases bearing either electron-donating or electron-withdrawing substituents were also suitable, and high yields and enantioselectivities were obtained with both 0.1 and 0.01 mol % catalyst loading (Table 2, entries 5–9). Sterically hindered substrates were also viable, and high enantioselectivities were obtained with 0.01 mol % catalyst loading (Table 2, entries 10–13).

**Table 2:** Scope of the reaction with respect to Schiff bases^a^.

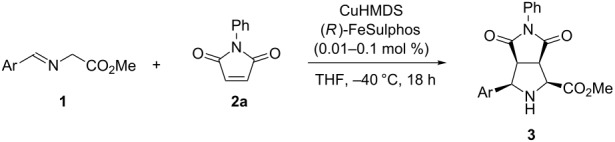							

Entry	Ar	**1**	FeSulphos (mol %)	**3**	Yield (%)	*Endo*/*exo*	ee (%, *endo*)

1	*p*-MeC_6_H_4_	**1b**	0.1	**3ba**	92	>99:1	>99
2	*p*-MeC_6_H_4_	**1b**	0.01	**3ba**	91	>99:1	85
3	*m*-MeC_6_H_4_	**1c**	0.1	**3ca**	93	>99:1	>99
4	*o*-MeC_6_H_4_	**1d**	0.1	**3da**	92	97:3	>99
5	*p*-MeOC_6_H_4_	**1e**	0.1	**3ea**	91	97:3	99
6	*p*-MeOC_6_H_4_	**1e**	0.01	**3ea**	91	>99:1	93
7	*p*-ClC_6_H_4_	**1f**	0.1	**3fa**	96	98:2	92
8	*p*-FC_6_H_4_	**1g**	0.1	**3ga**	96	>99:1	99
9	*p*-FC_6_H_4_	**1g**	0.01	**3ga**	96	>99:1	98
10	2-naphthyl	**1h**	0.1	**3ha**	94	>99:1	99
11	2-naphthyl	**1h**	0.01	**3ha**	87	>99:1	97
12	1-naphthyl	**1i**	0.1	**3ia**	76	>99:1	99
13	1-naphthyl	**1i**	0.01	**3ia**	91	>99:1	98

^a^Reaction conditions: For 0.1 mol % catalyst loading: the [3 + 2] cycloaddition reactions of 0.5 M **1** (10 mmol) with **2a** (11 mmol) were conducted at −40 °C for 18 h by using the chiral copper amide prepared from CuOTf·0.5toluene complex (0.011 mmol), KHMDS (0.010 mmol), and FeSulphos (0.011 mmol) in situ. For 0.01 mol % catalyst loading: the [3 + 2] cycloaddition reactions of 1.25 M **1** (50 mmol) with **2a** (55 mmol) were conducted at −40 °C for 48 h by using the chiral copper amide prepared from CuOTf·0.5toluene complex (0.0055 mmol), KHMDS (0.0050 mmol), and FeSulphos (0.0055 mmol) in situ.

**Scheme 1:**
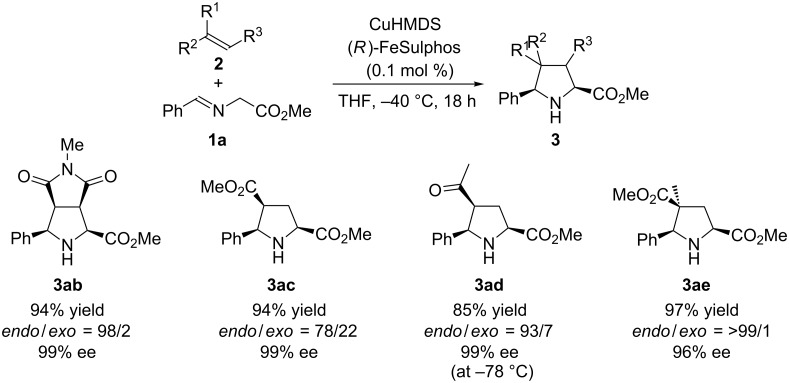
Scope of the reaction with other electrophiles^.^ The [3 + 2] cycloaddition reaction of 0.5 M **1a** (10 mmol) with **2** (11 mmol) was conducted at −40 °C for 18 h by using the chiral copper amide prepared from CuOTf·0.5toluene complex (0.011 mmol), KHMDS (0.010 mmol), and FeSulphos (0.011 mmol) in situ.

Other electrophiles were also successfully employed with 0.1 mol % catalyst loading (Scheme 1). *N*-Methylmaleimide reacted with **1a** in high yield with high diastereo- and enantioselectivities. The reaction with methyl acrylate also proceeded in high yield with high enantioselectivity; however, in this case the *exo*/*endo* selectivity was moderate. Methyl vinyl ketone and methyl methacrylate reacted with **1a** to afford the desired [3 + 2] adducts in high yields with high selectivities. Notably, the chiral CuHMDS catalyst worked well with catalyst loadings of both 0.1 and 0.01 mol %.

**Figure 1:**
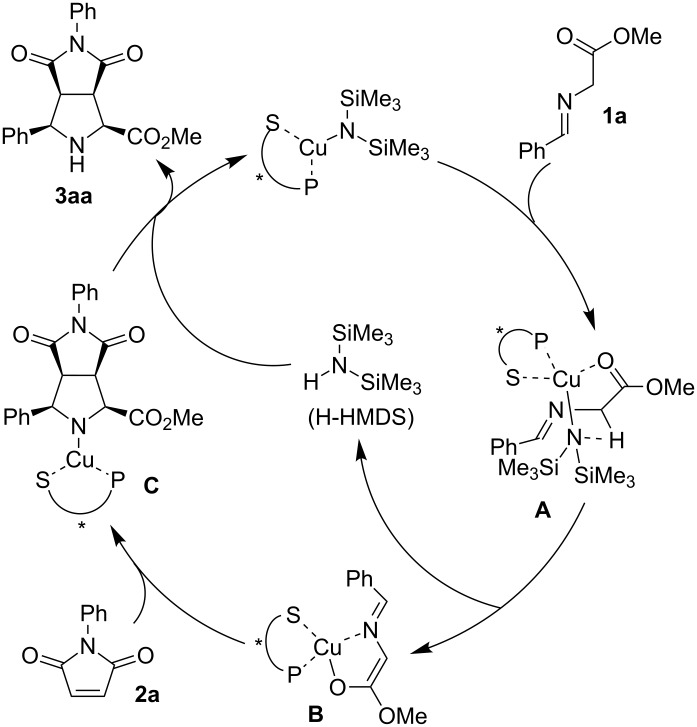
Proposed catalytic cycle.

A proposed catalytic cycle is shown in Figure 1. Thus, the chiral CuHMDS deprotonates Schiff base **1a** to generate the corresponding chiral Cu enolate **B** through the efficient formation of pseudo-intramolecular transition state **A**. Intermediate **B** reacted with maleimide **2a** to form Cu-pyrrolidine intermediate **C**. H-HMDS then reacted with the latter to regenerate the chiral CuHMDS and release the product to complete the catalytic cycle. The result obtained by using a mesitylcopper catalyst suggests that the reaction could also proceed through a product base mechanism in which the Cu-pyrrolidine intermediate **C** deprotonates the Schiff base **1a** directly; however, the higher reactivity observed upon catalysis by CuHMDS and the basicity of the intermediate indicates that the proposed cycle is reasonable when CuHMDS is used as catalyst. The high catalyst turnover may be due to the stronger Brønsted basicity of CuHMDS, which enables rapid deprotonation of the Schiff base.

## Conclusion

In conclusion, highly efficient asymmetric [3 + 2] cycloadditions catalyzed by chiral CuHMDS have been described. Compared with catalysis by using the CuOTf/Et_3_N system, the Cu amide system showed higher reactivity, and the reactions proceeded with high enantioselectivities even with 0.01 mol % catalyst loading. Further investigations that are focused on the application of metal amide catalysts in other reactions are ongoing.

## Experimental

A general experimental procedure for conducting catalytic asymmetric [3 + 2] cycloaddition reactions with 0.01 mol % catalyst loading is described. Under an Ar atmosphere, a solution of the preformed chiral CuHMDS catalyst [prepared from KHMDS (1.0 mg, 0.0050 mmol), CuOTf·0.5toluene (1.3 mg, 0.0055 mmol) and FeSulphos (2.3 mg, 0.0050 mmol) in anhydrous THF (5 mL) with heating at 40 °C for 1 h] was transferred into a well-dried 50 mL single-necked flask attached to a three-way cock (sealed with grease). The solution was cooled at −40 °C, and a mixture of **1** (50 mmol) and **2a** (55 mmol) in anhydrous THF (35 mL) was added by using a cannula. The whole was stirred for 48 h at −40 °C, then the reaction was quenched by the addition of H_2_O, and the mixture was extracted with dichloromethane. The organic layers were combined and dried over anhydrous Na_2_SO_4_. The selectivities were determined by ^1^H NMR analysis and HPLC analysis after purification of a small amount of the separated crude solution. After filtration and concentration under reduced pressure, the crude product obtained was purified by recrystallization and column chromatography to determine the isolated yield of the desired product. Obtained compounds were characterized by ^1^H and ^13^C NMR and by HPLC analyses using HPLC with chiral columns. The physical data for the products were consistent with reported values [49–54].
